# Rumen fermentation shifts and microbial dynamics in mid-lactating Holstein dairy cows experiencing heat stress and subsequent recovery periods

**DOI:** 10.5713/ab.24.0569

**Published:** 2024-10-28

**Authors:** Ye Pyae Naing, Seon-Ho Kim, A-Rang Son, Michelle Miguel, Joonpyo Oh, Sang-Suk Lee

**Affiliations:** 1 Ruminant Nutrition and Anaerobe Laboratory, Department of Animal Science and Technology, Sunchon National University, Suncheon 57922, Korea; 2 Cargill Animal Nutrition, Seongnam 13630, Korea

**Keywords:** Dairy Cows, Heat Stress (HS), Microbiota, Mid-Lactation, Recovery (RC), Rumen

## Abstract

**Objective:**

In this study, we investigated the effects of heat stress (HS) on rumen fermentation, blood parameters, and ruminal microbial communities in mid-lactating Holstein dairy cows in Korea.

**Methods:**

Our study involved 12 mid-lactation Holstein dairy cows aged 55.54 months with 2.5±0.65 parities and 100 to 200 days in milking (DIM), fed a total mixed ratio diet. Samples were collected during HS (temperature-humidity index [THI] = 81.69) and recovery (RC) period (THI 69.84). The samples were analyzed for rumen fermentation, blood parameters, heat shock proteins, and microbial communities in dairy cows.

**Results:**

The milk yield, milk fat, milk protein, and milk urea nitrogen levels differed significantly between two-time points (p<0.05). Rumen pH and volatile fatty acid concentrations, the pH was not significantly different (p = 0.619) between HS and RC periods; however, the ammonia nitrogen (NH_3_-N) levels increased during HS period ), however, there was no significant difference (p>0.05). Blood total protein significantly increased during HS period compared with that during RC period (p<0.05), while no significant differences were observed in other parameters between the two periods. HSP27, HSP70, and HSP90 increased in dairy cows under HS conditions compared with those during the RC period. Taxonomic classification revealed that *Firmicutes* and *Bacteroidetes* dominated the bacterial community. PERMANOVA and PERMDISP showed significant differences in rumen bacterial diversity between HS and RC periods, based on Unifrac metrics (p = 0.044 and p = 0.015, respectively), indicating taxonomic variations. Microbial networks with correlations of >0.8 (p<0.05) showed a complex structure with equal positive and negative connections, indicating *Anaerohabdus furcosa* and *Ruminiclostridium cellobioparum* as key species during the HS and RC periods respectively.

**Conclusion:**

HS significantly impacts Holstein dairy cows’ physiological and metabolic processes, altering rumen fermentation, blood biochemistry, and gut microbiota during mid-lactation.

## INTRODUCTION

Heat stress (HS) in animals occurs when the external temperature increases above the thermoneutral zone [1]. HS can severly injure dairy animals, and the dairy industry is particularly vulnerable to its negative impacts [2]. When evaluating the impact of HS on dairy cows, the temperature-humidity index (THI), an indicator that considers both ambient temperature (°C) and relative humidity (%), is frequently used [3]. When the ambient temperature or THI increases above 32.2°C or 68, respectively, physiological responses to HS become evident [4]. Several indicators are negatively affected by HS, including growth, dry matter intake, milk production, and the prevalence of health issues [5]. Animals have various physiological, endocrine, and behavioral mechanisms to deal with HS. In general, a decrease in feed intake is a significant indicator of negative energy balance, which can lead to reduced milk production [6]. The most common physiological response to HS is increased heat loss to maintain a constant body temperature [7]. Furthermore, the physiological responses of animals to HS during the breeding season affect several biochemical blood parameters [8].

The rumens of dairy cows are inhabited by an array of microorganisms essential for metabolism and general health [6]. Despite the increasing trend of temperature in the Republic of Korea owing to climate change, which is sensitive to elevated temperatures and undergoes thermal stress when the surrounding temperature rises above 27°C [9]. The rumen microbiome can be significantly affected by HS, and much is known about its effect on dairy cows during the transition from summer to winter during mid-lactation. Our research examined the consequences of how HS on Holstein dairy cows in Korea during their mid-lactation period. We investigated various aspects, including blood parameters, rumen microorganisms, rumen function, milk production, and physiological responses. While previous studies have primarily concentrated on blood parameters and bacterial communities, there is a scarcity of comprehensive research exploring the full range of HS effects on Holstein dairy cows during this specific lactation stage. In this study, we examined the effects of HS on various aspects of mid-lactating Holstein dairy cows in Korea, including blood parameters, rumen microorganisms, rumen function, milk production, and physiological responses.

## MATERIALS AND METHODS

### Animal care

All experimental procedures were conducted in accordance with the Animal Experimental Guidelines provided by the Sunchon National University Institutional Animal Care and Use Committee of the Republic of Korea. The experimental protocol was approved by SCNU-IACUC (approval number: SCNU IACUC-2018-01).

### Animals, diets, and experimental design

The experiments were conducted on lactating Holstein cows at mid-lactation (n = 12; average age of 55.54 months and 2.5±0.65 parities; 100 to 200 days) located in Suncheon-si, Jeollanam-do. The chosen periods for evaluating HS and recovery (RC) in mid-lactating Holstein dairy cows was during summer (first week to third week of August), with a large number of heat index days that can induce stress, followed by post-stress RC periods (first week to third week of September), were intended to capture the peak physiological sensitivity of the cows. During the experimental period, dairy cows were fed twice daily (06:00 and 17:00) with the total mixed ratio (TMR) diet, intended for mid-lactating cows, and milked twice (05:00 and 16:00) daily using milking machine. The cows were 166.50±8.06 days in milk (DIM) during HS period and 194.50±8.06 DIM during RC period.

The THI was closely monitored in the barn during the HS and RC periods. The following formula was used to calculate the THI during HS and RC periods THI [10].


THI=(0.8×ambient temperature)+[(% relative huminidity/100)×(ambient temperature-14.4)]+46.4.

In the first two weeks, the average THI values recorded in the barn during the HS and RC periods were 81.69 (temperature: 29.96°C; humidity: 72.76%), and 69.84 (temperature: 22.47°C; humidity: 70.01%), respectively. The THI was also monitored during the sampling on the third week HS and RC period.

### Sample collection

Rumen fluid and blood samples were collected 3 h before afternoon feeding for the analysis of basal diet ingredients. The chemical composition of the TMR is presented in Table 1. Rumen fluid was obtained from 12 Holstein dairy cows using stomach tubing and carefully placed in individual 50-mL conical centrifuge tubes, which were then stored in an ice chest. Blood samples were collected from the jugular veins of the animals for the analysis of blood parameters. Approximately 5 mL of the collected blood was transferred to a vacutainer tube for further analysis of blood chemical composition and complete blood count (CBC). The BD Vacutainer K2 EDTA 5.4 mg (Becto Drive, Franklin Lakes, NJ, USA) and BD Vacutainer SSRTM II Advance (Belliver Industrial Estate, Plymouth, UK) containers were used for this purpose. The blood samples were immediately placed in an ice box and transported to the laboratory for further processing.

### Analysis of rumen fermentation parameters

The pH of each sample was immediately measured using a pH meter (Seven Compact pH/Ion Meter S220; Mettler Toledo, Greifensee, Switzerland). Rumen fluid samples collected from each steer were divided into three equal parts and stored at −80°C for the subsequent analysis of ammonia nitrogen (NH_3_-N), volatile fatty acids (VFAs), and rumen microbiota. The NH_3_-N concentration was determined using a colorimetric method using a Libra S22 spectrophotometer (CB40FJ; Biochrom Ltd., Cambourne, UK), following a previously described protocol [11]. The VFA concentration was measured using high-performance liquid chromatography (HPLC; Agilent Technologies 1200 series; Agilent Technologies, Waldbronn, Baden-Wurttemberg, Germany). An ultraviolet (UV) detector (set at 210 and 220 nm), METACARB87H column (Varian, Palo Alto, CA, USA), and buffered solvent (0.0085 N H2SO4) at a flow rate of 0.6 mL/min were used for the HPLC analysis.

### Blood biochemistry, complete blood count, and heat shock protein analysis

To separate blood serum, blood samples were centrifuged at 4°C for 10 min at 4,000 rpm, resulting in a supernatant that was transferred to a new tube and stored at −20°C until analysis. The Catalyst OneTM Chemistry Analyzer (IDEXX Laboratories, Inc., Westbrook, ME, USA) was used to evaluate the blood serums for various parameters, including aspartate aminotransferase (AST), blood urea nitrogen (BUN), calcium (CA), cholesterol (CHOL), magnesium (MG), inorganic phosphate (PHOS), total bilirubin (TBIL), and total protein (TP), as well as glucose and β-ketone levels using the FreeStyle Optium Neo H kit from Precision Xtra Abbott. Additional blood serum samples were sent to the Pharmacy Department of Sunchon National University in Korea for metabolomic analyses. The collected blood samples were immediately transported to the laboratory for CBC analysis using an IDEXX ProCyte Dx Hematology Analyzer (INDEXX Laboratory, Inc.). The analyzer measured various parameters, including red and white blood cell (WBC) counts, hemoglobin concentration, hematocrit value, mean corpuscular volume (MCV), mean corpuscular hemoglobin (MCH), mean corpuscular hemoglobin concentration (MCHC), red cell distribution width (RDW), reticulocyte count, platelet count, mean platelet volume (MPV), platelet distribution width (PDW), and immature granulocyte count (IGC). Serum samples were analyzed for heat shock protein (HSP)27, HSP70, and HSP90 using commercially available enzyme-linked immunosorbent assay (ELISA) kits specific for bovine serum obtained from MyBioSource, Inc. (San Diego, CA, USA). The analysis was conducted in accordance with the manufacturer’s instructions.

### DNA extraction and metataxonomic analysis

The DNA from the rumen fluid samples of mid-lactating dairy cows at different HS and RC periods was extracted using the PowerSoil DNA Isolation Kit (Cat. No. 12888; MO BIO, Carlsbad, CA) and sent to Macrogen, Inc. (Seoul, orea) for metabolomic analysis of the rumen microbiota. The extracted DNA was measured using PicoGreen, and the quantity was determined using NanoDrop. An amplicon library was created for each sample using the Illumina 16S Metagenomic Sequencing Library protocols. The V3–V4 region of the 16S rRNA genes was amplified using a step-polymerase chain reaction (PCR) with primers Bakt_341F (5′-AGATGTGTATAAGAGACAG-3′) and Bakt_805R (5′-GATGTGTATAAGAGACAGG-3′). Multiplexing indices and Illumina sequencing adapters were added for ten cycles to the second PCR cycle. The amplicon libraries were normalized and their quantities were measured using PicoGreen. The size of the libraries was verified using a TapeStation DNA ScreenTape D1000 (Agilent). The individuals were combined in an equal molar ratio, sequenced on the MiSeq system (Illumina, San Diego, CA, USA) using a 2×300-bp, and subsequently trimmed using Trimmomatic (v0.38). The FLASH (1.2.11) program was used to join the two reads and sequences shorter than 400 bp were removed. We used rDnaTools to identify and eliminate chimeric sequences and subsampled samples to an equal depth of 10,000 sequences per sample to prevent bias caused by varying sequencing depths. The sequences were filtered for quality and subsequently clustered into operational taxonomic units (OTUs) with 97% sequence similarity using CD-HIT-OTUs. The representative sequences of each OTU were compared using the BLASTN (v2.4.0) tool of the Nucleotide Basic Local Alignment Search Tool against the 16S Microbial DB of the National Center for Biotechnology Information (NCBI) for taxonomic classification.

### Statistical analysis

Data on rumen fermentation, blood, and milk composition parameters between the two periods of analysis were analyzed using Statistical Analysis Systems (SAS) software version 9.4 (SAS Institute 2012). Data were statistically evaluated using Proc Glimmix for a completely randomized design using, analysis of variance (ANOVA) and t-tests. Duncan’s multiple range test was applied to determine the differences between the two periods, with p<0.05 indicating a statistically significant difference in the means.

The microbiome-related analyses were conducted as follows. Microbial diversity was estimated by calculating alpha diversity indices, such as the observed number of OTUs, Chao1, Shannon, and Simpson indices. Each metric was compared using GraphPad Prism 8.0.2 (263) test. Beta diversity analysis for dissimilarities in community structure was conducted using ANOVA, permutational multivariate ANOVA (PERMANOVA), and permutational analysis of multivariate dispersions (PERMSISP) analyses, which are statistical methods commonly used in ecological studies to analyze differences in the community composition and dispersion and further discriminate between HS and RC periods. The Bray-Curtis distance was used as the distance matrix. The microbial composition was visualized using stacked bar plots to depict the relative abundance of taxa at the phylum and species levels. Linear discriminant analysis effect size (LEfSe) was used to identify the biomarkers associated with the HS and RC periods. The interactions between ruminal species and the potential effects of the HS and RC periods on these connections and microbial co-occurrence networks were investigated.

## RESULTS

### Milk yield and composition

The significant differences in milk yield and composition between Holstein cows during the HS and RC periods are presented in Table 2. The milk yield, milk fat, milk protein, and milk urea nitrogen (MUN) levels differed significantly between HS (26.83±0.89 L/day, 3.75±0.03%, 3.00±0.08%, and 13.32±0.67 mg/day, respectively) and RC (32.73±0.89 L/day, 3.97±0.01%, 3.32±0.01%, and 15.43±0.77 mg/day, respectively), whereas solid non-fat (SNF) levels remained unchanged between HS (8.72±0.05%) and RC (8.85±0.05%). Milk yield and milk composition were higher in cows during the RC than in the HS period.

### Rumen fermentation parameters

**Figure 1:**
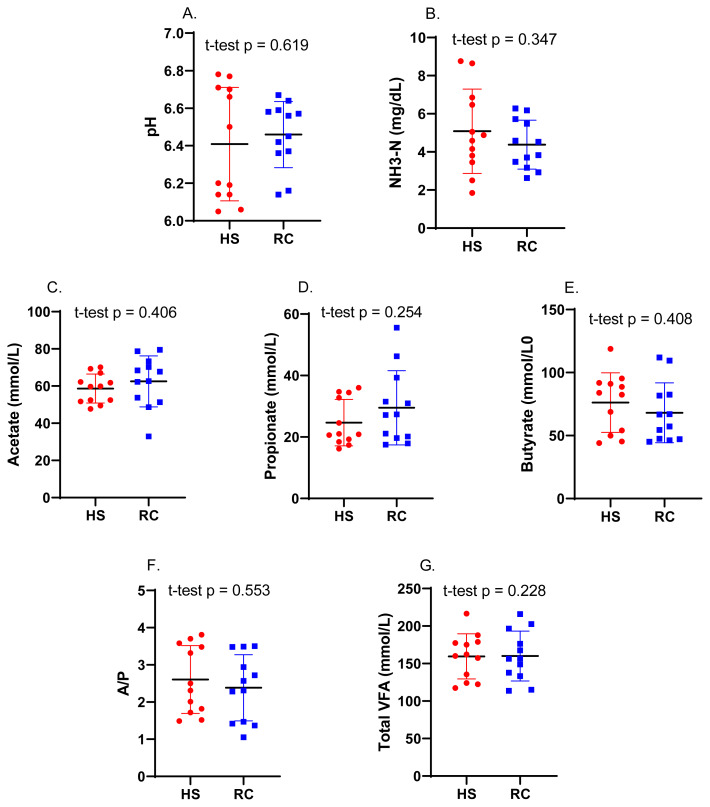
Rumen fermentation parameters. (A) pH, (B) NH_3_-N, (C) acetate acid, (D) propionate acid, (E) butyrate acid, (F) acetate to propionate ratio (A/P), and (G) total VFA, between heat stress (HS) and recovery (RC) periods. Numbers have been annotated for clarity. VFA, volatile fatty acids. A significant difference between HS and RC is indicated when (p<0.05).

The rumen fermentation parameters of Holstein cows during mid-lactation in the HS and RC periods are shown in Figure 1. Although rumen pH was slightly lower during HS, the pH was not significantly different (p = 0.619) between the HS (6.41±0.09) and RC (6.46±0.05) periods (Figure 1A). The NH_3_-N concentration increased during the HS period (5.09±0.64 mg/dL) compared with that during the RC period (4.38±0.37 mg/dL) (Figure 1B). Concentrations of acetate, propionate, and butyrate showed no significant difference between the HS (58.65±2.25, 24.69±2.18, and 76.21±6.81 mmol/L, respectively) and RC (62.51±3.95, 29.51±3.49, and 68.05±6.86 mmol/L, respectively) periods (Figures 1C–1E). Similarly, the acetate to propionate (A/P) ratio and total VFAs were not significantly different between the HS (2.61±0.26 and 159.56±8.70, respectively) and RC (2.38±0.26 and 160.06±9.59, respectively) (Figures 1F, 1G).

### Blood biochemistry, complete blood count, and heat shock protein analysis

**Figure 2:**
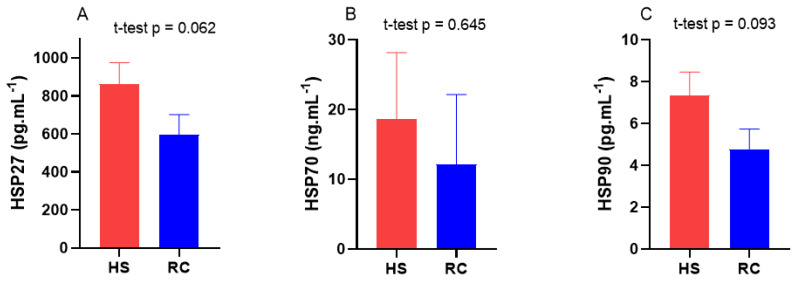
Blood serum concentrations of (A) HSP27, (B) HSP70, and (C) HSP90 between the heat stress (HS) and recovery (RC) periods. There is a significant difference between HS and RC when the p-value is less than 0.05 (p<0.05).

Blood serum biochemistry during the HS and RC periods is presented in Table 3. Blood glucose was lower (p = 0.063) during HS (52.83±1.04 mg/dL) compared with that during RC (55.33±0.74 mg/dL). Similarly, blood serum ketone concentration was higher (p = 0.651) during HS (0.83±0.07 mmol/L) compared with that during RC (0.78±0.09 mmol/L). BUN was higher in HS (12.42±0.76 mg/dL) than in RC (11.75±0.54 mg/dL), however, there was no significant difference (p = 0.483). Blood mineral levels (phosphorus, CA, and MG) showed no significant difference (p>0.05) between the HS (5.61±0.14, 8.80±0.10, and 2.34±0.05 mg/dL, respectively) and RC (5.83±0.16, 8.93±0.09, and 2.23±0.06, respectively) periods. In contrast, TP increased significantly (p = 0.012) in HS (8.44±0.11 g/dL) compared with that in RC (8.02±0.11 g/dL). Levels of AST, TBIL, and CHOL were not significantly different (p>0.05) between the HS (78.42±3.72 U/L, 0.38±0.02, and 198.83±8.01 mg/dL, respectively) and RC (83.08±3.67 U/L, 0.36±0.02, and 205.50±7.87 mg/dL, respectively) periods. During the HS period, there was a significant increase (p<0.05) in the red blood cell (RBC) count, hematocrit, hemoglobin, and platelet count (Table 4). However, these values decreased during the RC period. Moreover, significant changes in differential leukocyte counts and platelet indices were observed. The blood serum levels of HSP27, HSP70, and HSP90 remained constant during both HS (860.00±115.12 pg.mL^−1^, 18.61±9.58 ng.mL^−1^, and 7.36±1.11 pg.mL^−1^, respectively) and RC (714.53±84.09 pg.mL^−1^, 16.20±13.27 ng.mL^−1^, and 6.32±0.75 pg.mL^−1^, respectively). The levels of these proteins were higher but not significant (p>0.05) during the HS period compared with those during the RC period (Figures 2A–2C).

### Ruminal bacterial diversity

**Figure 3:**
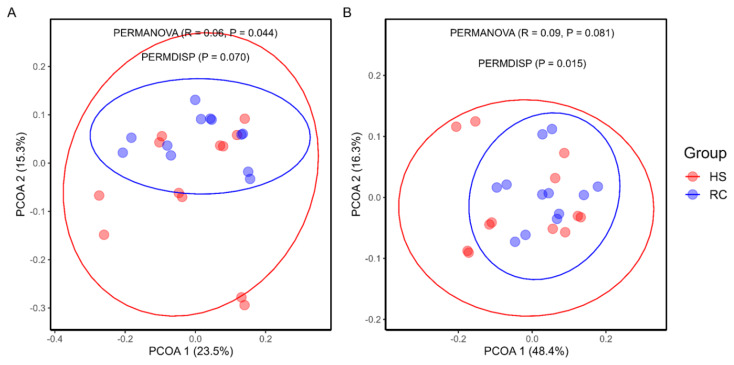
Beta diversity of the rumen bacterial community during the heat stress (HS) and recovery (RC) periods. PERMANOVA revealed significant beta diversity differences between HS and RC groups (p = 0.044) with unweighted UniFrac, and PERMDISP showed significant variation (p = 0.015) with weighted UniFrac. PCoA, principal coordinate analysis; PERMDISP, permutational multivariate analysis of variance.

Microbial diversity varied based on the beta and alpha diversity metrics, revealing variations in the microbial communities. Principal coordinate analysis (PCoA) using unweighted and weighted UniFrac distances (Figures 3A,3B) was conducted to assess the dissimilarities in the bacterial communities. PERMANOVA of the rumen bacterial community revealed a significant difference (p = 0.044) in the beta diversity between the HS and RC groups based on unweighted UniFrac distances. Weighted UniFrac, which incorporates both the presence and abundance of taxa, showed significant variation (p = 0.015) in the beta diversity based on PERMDISP analysis.

**Figure 4:**
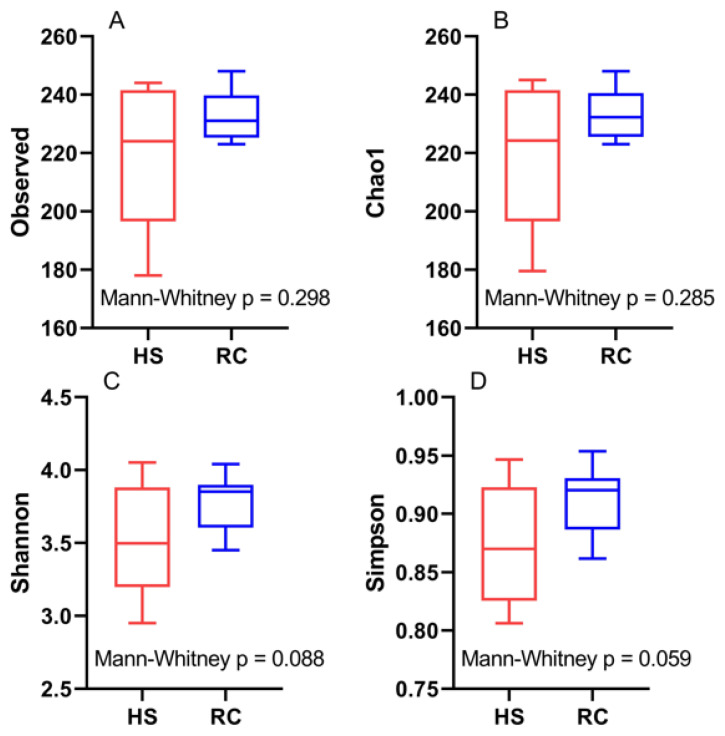
Boxplot representation of the alpha diversity index. (A) observed, (B) Chao1, (C) Shannon, (D) Simpson indices between the heat stress (HS) and (RC) periods. The visualization of alpha-diversity metrics was conducted in MicrobiomeAnalyst. There is a significant difference between HS and RC when the p-value is less than 0.05 (p<0.05).

The results of the alpha diversity index analysis showed no significant differences between the HS and RC groups in terms of the observed OTUs, Chao1, Shannon, and Simpson indices (Figures 4A–4D).

**Figure 5:**
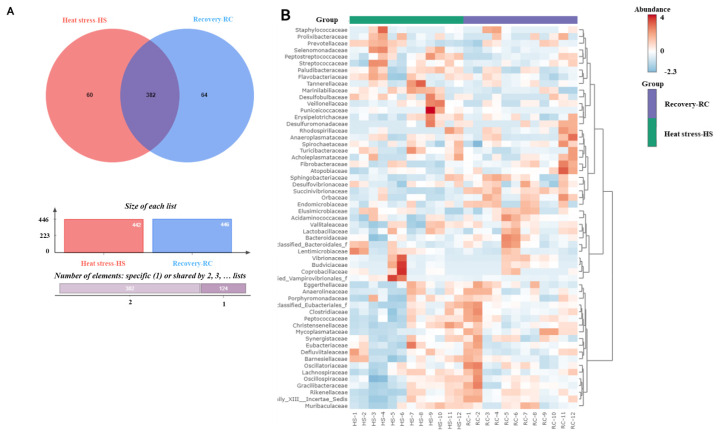
The representation of the differences in the bacterial communities. (A) Venn diagram of rumen bacteria in the heat stress (HS) and recovery (RC) periods, both groups shared 382 species, with 60 species unique to HS and 64 to RC. (B) Hierarchical clustering heatmaps analysis in the HS and RC periods. The bar graph below depicts the size of representative species of observed operational taxonomic units (OTUs) by period. The top of the heatmap shows HS in green and RC in purple. The relationship between bacterial communities abundance and periods is shown by the color spectrum of red to blue, indicating high to low abundance.

The presence of 382 shared species within both the HS and RC groups, along with 60 and 64 exclusive species in the HS and RC gruups, respectively, was analyzed using heatmap analysis to more effectively compare the variations in ruminal bacterial communities between the HS and RC periods. In the analysis, we focused on the top 57 bacterial communities and revealed statistically significant differences between these periods. These results are shown in Figures 5A, 5B, which shows a graphical representation of the differences in the bacterial communities under these conditions.

### Ruminal bacterial composition

**Figure 6:**
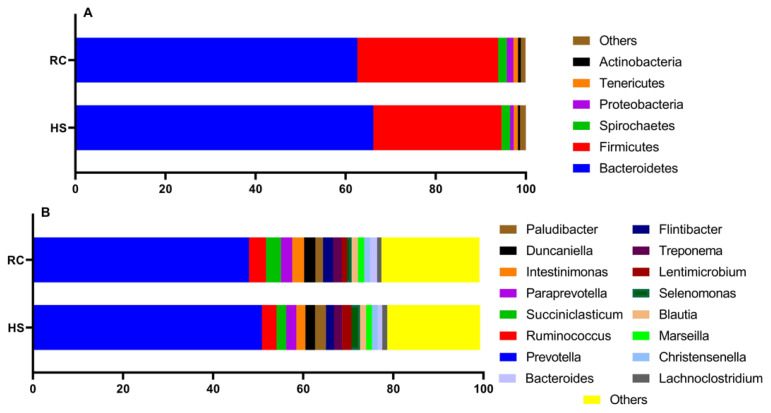
Mean ruminal taxonomic profiles at the (A) phylum level are associated with the heat stress (HS), and recovery (RC) periods. The stacked bar plots represent the top 10 phyla. (B) Genus level associated with the HS, and RC periods. The stacked bar plots represent the top 20 species. An average relative abundance of ≥2%.

**Figure 7:**
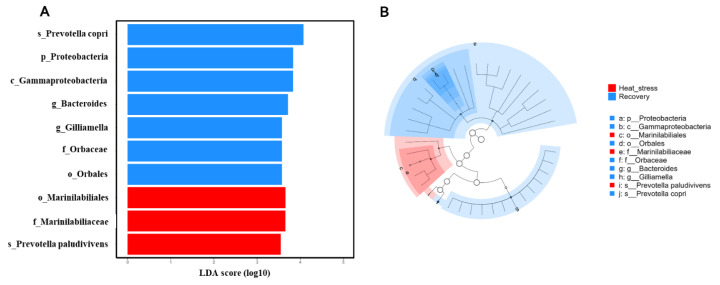
Linear discriminant analysis effect size (LEfSe) was used to determine the effect size of heat stress (HS) on the rumen bacterial community during the recovery (RC) period. (A) The differential bacterial changes with their corresponding log 10-transformed effect sizes (LDA scores) represented by the length of the bar. The colors used in the figure indicate the group in which the taxa were more abundant compared with the other group. (B) Cladogram displaying the differential bacteria in a phylogenetic tree, wherein the color rrepresents the branch most significantly representing a particular group.

**Figure 8:**
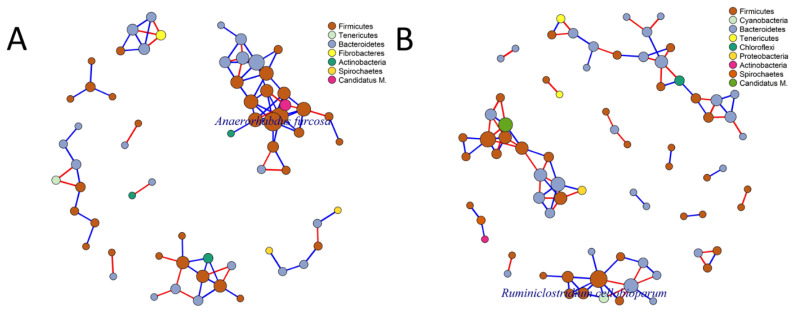
The microbial co-occurrence network of ruminal microbiota of mid-lactation dairy cows during the (A) heat stress (HS) and (B) recovery (RC) periods. The network is used to describe microbial interactions and patterns of samples belonging to the HS and RC periods. Prevalence filtering was set at 0.75, followed by the selection of strong correlations with an absolute value of 0.8 and a p-value below the threshold of 0.05.

In this his study, we aimed to determine whether HS during mid-lactation affects the composition of ruminal microbiota. Ruminal bacteria were taxonomically classified at the phylum and genus levels to compare the relative abundances of the microbiota composition. Based on the taxonomic analysis of the reads, three predominant phyla (with an average relative abundance of ≥2%) were identified in the rumen during the HS and RC periods: *Bacteroidetes, Firmicutes*, and *Spirochaetes*. At the genus level, ten predominant genera (with an average relative abundance of ≥2%) were identified in the same period, including *Prevotella, Ruminococcus, Succiniclasticum, Paraprevotella, Intestinimonas, Duncaniella, Paludibacter, Flintibacter, Treponema*, and *Lentimicrobium*, as shown in Figures 6A, 6B. LEfSe was conducted to determine the singular effect of the HS and RC periods on the ruminal microbiota. During the HS, *Marinilabiliales, Marinilabiliaceae*, and *Prevotella paludivivens* were enriched, whereas during the RC period, seven taxa were enriched: *Prevotella copri, Proteobacteria, Gammaproteobacteria, Bacteroides, Gilliamella, Orbaceae*, and *Orbales*, as shown in Figures 7A, 7B. To investigate the interactions between ruminal species and the potential effects of the HS and RC periods on these connections, microbial co-occurrence networks were constructed. Prevalence filtering was set at 0.75, followed by the selection of strong correlations with an absolute value of 0.8 and a p-value below the threshold of 0.05. For instance, the network constructed from the HS and RC samples was more complex, with 63 species interconnected by 91 edges (connections). Similar numbers of negative and positive connections were observed. A higher number of microbial phyla was also observed, with the co-dominance of *Firmicutes* and *Bacteroidetes*. *Anaerorhabdus furcosa* was identified as a keystone species with seven connections during the HS. A total of 72 species were interconnected with 92 edges (connections). Similar numbers of negative and positive connections were observed. A higher number of microbial phyla was also observed, with the co-dominance of *Firmicutes* and *Bacteroidetes*. *Ruminiclostridium cellobioparum* was identified as a keystone species with nine connections during the RC period, as shown in Figures 8A, 8B.

## DISCUSSION

The dairy industry has long battled with the negative consequences of HS on dairy cows, which not only impairs their performance but also increase the risk of metabolic disorders and health issues. This problem is particularly acute in regions, such as Korea, where temperature fluctuations can be considerable. Mid-lactating Holstein dairy cows, a significant part of the dairy industry, are particularly susceptible to HS owing to their physiological state. Understanding the influence of HS on rumen fermentation, blood parameters, and rumen microbial communities in dairy cows is vital for devising effective management strategies [12].

HS significantly decreases milk production in mild-lactating Holstein cows by reducing feed intake. One study found that milk yield, milk fat, milk protein, and MUN differed significantly between the two-time points. However, SNF reduced by almost 10% during HS compared with that during the RC period, which is consistent with previous research [13]. The results also showed that milk yield increased in the early stage of lactation (≤100 DIM) compared with that during mid-lactation (101 to 200 DIM) by more than 10%, regardless of parity [14]. These findings suggest that HS can have a negative effect on milk production in mild-lactating The decrease in rumen pH during HS is typically attributed to an imbalance in the production and consumption of acids, as indicated by previous research [15]. To rectify this imbalance, appropriate management practices should be implemented during the RC to restore normal rumen pH levels [15]. This can be attributed to changes in the abundance and composition of acid-producing and acid-utilizing bacteria in the rumen [15]. For example, a previous study [15] indicatd that HS significantly decreased the NH_3_-N content. As a source of energy, the rumen bacteria of ruminants break down the diet and create VFA [16]. However, some studies have indicated that HS can change the proportions of butyrate and acetate in the rumen, with increasing and decreasing butyrate and acetate contents, respectively [17]. Moreover, HS can increase the fluid content and demand in the rumen, which can affect the VFA concentration [18]. However, one study found no significant differences in the A/P ratio and total VFA between the two -time points. Inconsistencies in the results regarding the A/P ratio and total VFAs during HS can be attributed to several interrelated factors including the severity and duration of HS, metabolic shifts in dairy cows, and individual responses based on physiological conditions.

Body glucose levels are affected by both the intensity and duration of HS. Previous studies by [19,20] support this notion. Body glucose levels depend on the severity and duration of HS. Dairy cows under HS may experience changes in glucose levels owing to reduced feed intake and an imbalance in energy, particularly carbohydrates. This can lead to energy depletion in cows that is not influenced by the lactation stage. The severity and duration of HS significantly impact glucose metabolism, feed intake, and overall energy balance in mid-lactating dairy cows. This alteration in nutrient availability can directly affect the rumen microbial population and its fermentation pattern, particularly the production of VFAs. In this study, HS increased blood ketone levels in mid-lactating Holstein cows. This is because HS can reduce feed intake, resulting in an energy imbalance and the release of body fat for energy. During the RC period, ketone levels may return to normal as cows resume regular feeding, and the energy balance is restored. The concentration of ketones in the blood serum significantly increases during HS, probably owing to an adaptive response from reduced energy intake and the net energy balance status [21]. The reduction in feed intake due to HS changes the nutrient availability for rumen microorganisms. As a result, the dynamics of rumen fermentation are altered, potentially affecting the concentrations of VFAs produced, which can also impact rumen pH levels [22]. Therefore, the variation in these fermentation patterns can be closely tied to the dietary changes experienced by heat-stressed cows. The stage of lactation plays a critical role in how dairy cows respond to HS. For example, an increase in blood ketone levels during periods of HS indicates a metabolic shift towards fat mobilization, which compensates for the reduced availability of carbohydrates [6]. This shift alters the rumen environment by changing the substrates available for fermentation, potentially leading to different types of VFA production patterns and variations in rumen pH [23]. The relationship between microbial dynamics and rumen fermentation becomes apparent when considering how specific bacterial populations adapt to changes in the rumen environment. Bacteria responsible for carbohydrate fermentation, such as cellulolytic bacteria, tend to be more sensitive to fluctuations in feed intake and energy supply, affecting both VFA concentration and rumen pH [24]. A decline in feed intake during HS diminishes the availability of fermentable carbohydrates, which can result in reduced VFA production and less significant changes in rumen pH. In this study, HS decreased blood phosphorus and CA levels in mid-lactating Holstein cows. HS can result in decreased feed intake, and phosphorus is an essential nutrient for milk production. Previous studies have demonstrated that phosphorus and CA levels decreased at high ambient temperatures [25]. Conversely, the blood magnesium levels increased during the HS period. A lower TP concentration during mid-lactation indicates undesired protein efficiency in dairy cows at later stages [26]. We found a decrease in blood AST levels in mid-lactation Holstein cows during the HS period compared with the RC periods. HS can increase blood AST levels in mid-lactating Holstein cows owing to liver damage. AST levels are known to decrease during RC as the liver heals and normal function is restored. An elevated serum AST activity is a sensitive marker of liver damage [27]. Additionally, a significant increase in the TBIL level was observed during the RC period. In agreement with this study, a previous study [27]. Found an increase in bilirubin concentration in the blood serum of cows under HS compared with that in the thermoneutral period. They found that dairy cows under HS had increased WBC counts compared with those under RC [28]. In this study, we also found that the RBC count decreased during the HS period compared with that during the RC period. HS can damage RBCs, resulting in a decrease in their counts and lower average RBC count. Hemoglobin (Hb) concentrations in dairy cows can increase owing to HS. After HS, dairy cows may require more time to recover before their CBC counts return to normal levels. This is because HS can cause severe damage; thus, the body may require time to recover. A decrease in WBC count during the RC period may indicate that the immune systems of dairy cows are functioning well. In this study, the serum levels of HSP27, HSP70, and HSP90 were higher during HS than during RC. Similarly, a previous study [29] reported that HS significantly elevated the serum concentrations of heat shock factor (HSF), HSP27, HSP70, and HSP90 in dairy cows. Thermal stress also increases the plasma concentration of HSP27 and HSP70 gene expression in Holstein skin [30].

Accoding to several studies, the diversity and richness of rumen microbiota are crucial components that can significantly influence rumen function [27,31]. In this study, PERMANOVA was conducted on the beta diversity of rumen bacterial communities, which revealed a statistically significant difference (p = 0.044) between the HS and RC periods using unweighted UniFrac. Furthermore, PERMDISP analysis showed considerable variation (p = 0.015) in beta diversity during the HS and RC periods when using weighted UniFrac. We examined the alpha diversity metrics, including abundance-based coverage estimator - observed species, Chao1, Shannon, and Simpson, for the HS and RC groups. Our analysis revealed no substantial differences between the two groups. Previous studies showed that alpha diversity remained similar and was not affected [32].

Previous studies identified *Proteobacteria, Firmicutes*, and *Bacteroidetes* as the three most dominant phyla in the rumen [5]. We also found that these three phyla were most prevalent in the rumen of dairy cows during mid-lactation in both the HS and RC periods. HS affected the relative abundances of various genera at the species level. *Prevotella* exhibits a range of capabilities in the rumen, such as the degradation of hemicelluloses, pectinolysis, and proteolysis [33]. The ability of this microorganism to adapt to diverse conditions allows it to play a versatile role in digestive processes. *Ruminococcus*, known for its ability to degrade cellulose, exhibits low abundance in the gut microbiome during HS [34]. Despite this, *Ruminococcus* species play a key role in cellulose degradation in the rumen, with some species capable of fermenting starch, which contributes to their higher abundance during periods of a high-concentrate diet [34,35]. Additionally, *Succiniclasticum* is prevalent in cows fed with high concentrations of sucrose, which plays a crucial role in converting succinate to propionate, a vital precursor of glucose for ruminants. This contributes to the maintenance of rumen homeostasis by reducing sulfate levels and increasing metabolic flexibility [36]. In contrast, *Bacteroides* species rely on soluble carbohydrates as their energy source [37] and is highly effective in breaking down structural carbohydrates [35]. The abundance of *Bacteroides* in the gut microbiome is also altered during HS [23].

The findings from the LEfSe revealed insights into the alterations in ruminal microbiota in response to HS and RC. During the HS period, certain taxa were enriched compared with those during baseline or control conditions. In particular, *Marinilabiliales, Marinilabiliaceae*, and *P. paludivivens* were enriched [38]. This suggests that these microbial taxa may have a competitive advantage or may be better adapted to HS conditions. *Marinilabiliales* and *Marinilabiliaceae* are families within the class of bacteria known as *Bacteroidetes*, whereas *P. paludivivens* belongs to the genus *Prevotella*, which is also within the phylum *Bacteroidetes*. During the RC period following HS, a distinct set of microorganisms was found to be more prevalent than during both HS period and baseline conditions. Seven of these microorganisms were enriched during the RC: *P. copri, Proteobacteria* (phylum), *Gammaproteobacteria* (class within *Proteobacteria*), *Bacteroides* (genus within *Bacteroidetes*), *Gilliamella* (genus within *Gammaproteobacteria*), *Orbaceae* (family within *Gammaproteobacteria*), and *Orbales* (order within *Gammaproteobacteria*) [38]. This suggests that ruminal microbiota dynamically responds to the cessation of HS and returns to more favorable conditions. *P. copri*, *Bacteroides*, and *Gilliamella* are commonly found in the rumen and play important roles in various metabolic processes related to digestion and fermentation. During the RC period, the increase in *Proteobacteria, Gammaproteobacteria*, and related taxa suggests a potential shift toward a more diverse microbial community, as *Proteobacteria* are known to have a wide range of metabolic functions and ecological niches. Microbial co-occurrence networks and related techniques are highly valuable in systems biology, as they enable researchers to infer community structures based on bacterial abundance [39]. Using these networks, scientists can provide insights into the structural composition of microbiomes and their response to environmental changes [40]. When studying microbial networks, various interactions between species can be observed through different types of correlations. Negative correlations often indicate competitive or exclusionary relationships between species, whereas positive correlations suggest symbiotic or mutualistic relationships [41]. In our analysis, we identified *A. furcosa* and *R. cellobioparum* as vital species during the HS and RC periods, respectively. Additionally, we observed a strong association between *Firmicutes* and *Bacteroidetes* in both periods. The relationship between these two phyla is crucial as their imbalanced abundance has been used as a biomarker for gut dysbiosis, a condition characterized by an imbalance in gut bacteria [42].

## CONCLUSION

The HS significantly affects the physiological and metabolic processes of mid-lactating Holstein cows. HS led to a notable shift in rumen fermentation parameters, blood biochemical profiles, and rumen microbiota. While VFA concentrations remained stable, significant alterations were observed in blood glucose, TP, ketone, and cholesterol levels, highlighting a metabolic shift under stress. The rumen microbiota exhibited distinct bacterial taxa dominating during HS and RCs. These findings highlight the interactions between HS, metabolism, and rumen microbial dynamics in dairy cows, offering insights into mitigating the negative impacts of HS on dairy production. However, samples should be collected at a specific RC time, such as the lower THI period, to assess the substantial effect of HS.

## Figures and Tables

**Table 1:** Chemical composition (%) of total mixed ratio (TMR) fed to lactating Holstein cows (DM basis)

Item	DM basis
DM	87.96
Crude protein	13.05
Crude fat	2.99
Crude fiber	15.30
Crude ash	6.10
Calcium	0.69
Phosphorus	0.26
ADF	18.90
NDF	36.78

DM, dry matter; ADF, acid detergent fiber; NDF, neutral detergent fiber.

**Table 2:** Milk yield and composition of Holstein cows during heat stress (HS) and recovery (RC) periods

Parameters	HS	RC	SEM	p-value
Milk yield (L/day)	26.83±0.43^b^	32.73±0.89^a^	0.661	<0.0001
Milk fat (%)	3.75±0.03^b^	3.97±0.01^a^	0.021	<0.0001
Milk protein (%)	3.00±0.08^b^	3.32±0.01^a^	0.044	0.0005
SNF (%)	8.72±0.05	8.85±0.05	0.052	0.0890
MUN (mg/day)	13.32±0.67^b^	15.43±0.77^a^	0.719	0.0498

SEM, standard error of the mean; SNF, solid non-fat; MUN, milk urea nitrogen.

a,b Means with different superscripts in the same row differ significantly (p<0.05).

**Table 3:** Effect of the heat stress (HS) and recovery (RC) periods on blood biochemical parameters

Parameters	HS	RC	SEM	p-value
Glucose (mg/dL)	52.83±1.04	55.33±0.74	0.889	0.063
Ketone (mmol/L)	0.83±0.07	0.78±0.09	0.076	0.651
Blood urea nitrogen (mg/dL)	12.42±0.76	11.75±0.54	0.651	0.483
Phosphorus (mg/dL)	5.61±0.14	5.83±0.16	0.150	0.321
Calcium (mg/dL)	8.80±0.10	8.93±0.09	0.096	0.336
Magnesium (mg/dL)	2.34±0.05	2.23±0.06	0.052	0.164
Total protein (g/dL)	8.44±0.11^a^	8.02±0.11^b^	0.110	0.012
Aspartate aminotransferase (U/L)	78.42±3.72	83.08±3.67	3.695	0.382
Total bilirubin (mg/dL)	0.38±0.02	0.36±0.02	0.018	0.338
Cholesterol (mg/dL)	198.83±8.01	205.50±7.87	7.940	0.559

SEM, standard error of the mean.

a,b Means with different superscripts in the same row differ significantly (p<0.05).

**Table 4:** Effect of the heat stress (HS) and recovery (RC) periods on complete blood count (CBC)

Parameters	HS	RC	SEM	p-value
RBC (M/μL)	5.38±0.03^b^	6.36±0.18^a^	0.108	<0.0001
HCT (%)	38.28±0.94^a^	28.76±0.97^b^	0.952	<0.0001
HGB (g/dL)	14.58±0.44^a^	10.04±0.26^b^	0.349	<0.0001
MCV (fL)	48.33±0.81	47.67±0.72	0.764	0.5492
MCH (pg)	15.88±0.21^a^	12.69±0.34^b^	0.272	<0.0001
MCHC (g/dL)	38.67±0.22^a^	33.24±0.29^b^	0.253	<0.0001
RDW (%)	14.33±0.33^a^	13.07±0.26^b^	0.298	0.007
RETIC (K/μL)	2.31±0.06^a^	1.04±0.09^b^	0.075	<0.0001
WBC (K/μL)	17.44±0.16^a^	11.31±0.39^b^	0.276	<0.0001
NEU (%)	69.01±2.55^a^	35.89±1.30^b^	1.929	<0.0001
LYM (%)	19.50±0.32^b^	36.18±1.28^a^	0.800	<0.0001
MONO (%)	21.89±2.07^a^	9.53±0.21^b^	1.142	<0.0001
EOS (%)	2.08±0.15^b^	4.43±0.13^a^	0.140	<0.0001
BASO (%)	0.14±0.03^b^	0.70±0.05^a^	0.041	<0.0001
NEU (K/μL)	6.91±0.21^a^	3.77±0.19^b^	0.200	<0.0001
LYM (K/μL)	2.69±0.16	2.32±0.50	0.332	0.4947
MONO (K/μL)	3.38±0.14^a^	2.49±0.40^b^	0.271	0.0491
EOS (K/μL)	0.26±0.04^b^	0.90±0.07^a^	0.059	<0.0001
BASO (K/μL)	0.01±0.00^b^	0.21±0.02^a^	0.014	<0.0001
PLT (K/μL)	478.17±18.77^a^	371.50±14.46^b^	16.616	0.0002
MPV (fL)	10.90±0.24^a^	9.02±0.22^b^	0.230	<0.0001
PDW (fL)	13.18±0.35^a^	10.23±0.19^b^	0.269	<0.0001
IGC (%)	0.52±0.01^a^	0.38±0.01^b^	0.012	<0.0001

SEM, standard error of the mean; RBC, total number of erythrocytes; HCT, hematocrit value: erythrocyte ratio of total blood volume; HGB, hemoglobin concentration; MCV, mean erythrocyte volume in total sample; MCH, mean hemoglobin volume per RBC count; MCHC, mean hemoglobin concentration of erythrocytes; RDW, degree of variation in size of the erythrocyte population; RETIC, reticulocyte count; WBC, total number of leukocytes; NEU, neutrophil percentage; LYM, lymphocyte percentage; MONO, monocyte percentage; EOS, eosinophil percentage; BASO, basophil percentage; NEU, neutrophil count; LYM, lymphocyte count; MONO, monocyte count; EOS, eosinophil count, BASO, basophil count; PLT, total number of platelets; MPV, mean platelet volume; PDW, platelet distribution width; IGC, immature granulocyte count.

a,b Means with different superscripts in the same row differ significantly (p<0.05).
